# Accuracy and Adoption of Wearable Technology Used by Active Citizens: A Marathon Event Field Study

**DOI:** 10.2196/mhealth.6395

**Published:** 2017-02-28

**Authors:** Monika Pobiruchin, Julian Suleder, Richard Zowalla, Martin Wiesner

**Affiliations:** ^1^ GECKO Institute for Medicine, Informatics & Economics Heilbronn University Heilbronn Germany; ^2^ Consumer Health Informatics SIG German Association for Medical Informatics, Biometry & Epidemiology (GMDS e.V.) Cologne Germany; ^3^ Department of Medical Informatics Heilbronn University Heilbronn Germany

**Keywords:** athlete, wearables, mobile phones, physical activity, activity monitoring

## Abstract

**Background:**

Today, runners use wearable technology such as global positioning system (GPS)–enabled sport watches to track and optimize their training activities, for example, when participating in a road race event. For this purpose, an increasing amount of low-priced, consumer-oriented wearable devices are available. However, the variety of such devices is overwhelming. It is unclear which devices are used by active, healthy citizens and whether they can provide accurate tracking results in a diverse study population. No published literature has yet assessed the dissemination of wearable technology in such a cohort and related influencing factors.

**Objective:**

The aim of this study was 2-fold: (1) to determine the adoption of wearable technology by runners, especially “smart” devices and (2) to investigate on the accuracy of tracked distances as recorded by such devices.

**Methods:**

A pre-race survey was applied to assess which wearable technology was predominantly used by runners of different age, sex, and fitness level. A post-race survey was conducted to determine the accuracy of the devices that tracked the running course. Logistic regression analysis was used to investigate whether age, sex, fitness level, or track distance were influencing factors. Recorded distances of different device categories were tested with a 2-sample *t* test against each other.

**Results:**

A total of 898 pre-race and 262 post-race surveys were completed. Most of the participants (approximately 75%) used wearable technology for training optimization and distance recording. Females (*P*=.02) and runners in higher age groups (50-59 years: *P*=.03; 60-69 years: *P<*.001; 70-79 year: *P*=.004) were less likely to use wearables. The mean of the track distances recorded by mobile phones with combined app (mean absolute error, MAE=0.35 km) and GPS-enabled sport watches (MAE=0.12 km) was significantly different (*P*=.002) for the half-marathon event.

**Conclusions:**

A great variety of vendors (n=36) and devices (n=156) were identified. Under real-world conditions, GPS-enabled devices, especially sport watches and mobile phones, were found to be accurate in terms of recorded course distances.

## Introduction

### Overview

Wearable technology such as global positioning system (GPS)-enabled sport watches, activity trackers, heart rate monitors, or even smart clothing is considered the number 1 trend in 2016 and 2017 according to the world-wide survey of fitness trends [1,2]. Mobile phones and related exercise apps are likewise ranked in the top 20 of this survey. Due to the ubiquitous nature of wearables and mobile phones, app features such as distance recording, optimization of training sessions, and the information on burned calories are no longer merely available for professional athletes. However, the variety of wearable devices for activity monitoring is overwhelming. The systematic research in terms of device or app accuracy in nonlaboratory settings in the context of long-distance running seems to be underrepresented in the literature [3].

### Related Work

According to Düking et al [4], wearables “are lightweight, sensor-based devices that are worn close to or on the surface of the skin, where they detect, analyze, and transmit information concerning several internal and external variables to an external device (...),” (p. 2). In particular, GPS-enabled devices can be considered reliable tracking devices, which holds true even for inexpensive systems.

As a study conducted by Pugliese et al suggests, the increasing use of wearables among consumers has implications for public health. Monitoring an individual’s personal activity level, for example, steps taken in one day, can result in an increased overall physical activity [5]. A moderate level of physical activity can prevent widespread diseases such as diabetes or hypertension [6-8] and thus result in decreasing costs for public health care systems in the long term [9,10].

Yet, in the context of the quantified-self movement, a high accuracy of these consumer-centric devices is desirable. In theory, the measurements obtained by different vendors and device categories (ie, GPS-enabled system vs accelerometer-based) should be comparable with each other [11].

Noah et al studied the reliability and validity of 2 Fitbit (Fitbit, San Francisco, CA) activity trackers with 23 participants. There seems to be evidence that these particular devices produce results “valid for activity monitoring” [12].

A study by Ferguson et al evaluated several consumer-level activity monitors [13]. The findings suggested the validity of fitness trackers with respect to measurement of steps; however, their study population was limited to 21 young adults.

At present, and to the best of our knowledge, no study exists that examines the adoption of consumer-level devices in a broad and diverse population. This is supported by the meta-analysis by Evenson et al: “Exploring the measurement properties of the trackers in a wide variety of populations would also be important in both laboratory and field settings.” We conclude that “more field-based studies are needed” (p. 20) [3]. In particular, this should include all age groups, different fitness levels, and a great variety of related devices.

### Aims of the Study

This study addressed the need for more real-life field evaluations of wearable devices [3]. This is especially important for researchers as well as for providers of health care programs. For instance, insurance companies offering reduced payments to their customers can thereby analyze the distribution of smart wearable devices and their respective accuracy. This allows for adjustments in health intervention programs. Moreover, the study provided a first baseline for researchers that want to validate their own findings in this field.

In this context, the aim of the study was 2-fold: (1) to determine the adoption of wearable technology, especially “smart” devices and (2) to investigate on the accuracy of tracked distances as recorded by such devices. The study cohort comprises participants from a public “Sport for All” road running event, that is, primarily physically active and healthy citizens across all age groups.

## Methods

### Road Running Event

The Trollinger-Marathon is an annual running event located in Heilbronn, a city in southern Germany [14,15]. In 2016, runners could choose between 4 different course distances: (1) full marathon, 42.195 km; (2) half-marathon, 21.0975 km; (3) walking or nordic walking course, 14.4 km; and (4) a marathon relay, approximately 3 × 14 km. The event itself took place on May 8, 2016. According to the organizer, a total of 6894 adult runners had registered for the event. Of the registered runners, 6481 actually lined up for the race of which finally 6331 completed the course [15]. The event organizer was a member of the German Road Races Society, and both the full marathon and half-marathon courses were measured according to Association of International Marathons and Road Races (AIMS) and International Association of Athletics Federation (IAAF) regulations. Both event categories were precisely measured by an accredited AIMS and IAAF Grade A or B measurer and therefore considered a valid baseline for the intended distance comparison.

At city marathon events, for example, New York or Berlin, GPS signal strength can be influenced by narrow streets and house constructions [16]. As the Trollinger-Marathon course is mainly characterized by an open landscape, no building-associated limitations exist at the event location. Thus, a good overall GPS coverage can be assumed.

### Questionnaire

Two questionnaires were designed: (1) a pre-race questionnaire, Q_1,_ to determine which kind of performance monitoring technology was predominantly used by runners of different age, sex, and fitness level and (2) a post-race questionnaire, Q_2,_ to determine the accuracy of the devices that tracked the running course.

Q_1_ consisted of 6 items by which quantitative and qualitative data were obtained (see Multimedia Appendix 1 for questions and response options). The primary aim of Q_1_ was the collection of cohort-specific data, that is, (1) age, (2) sex, (3) the devices used for exercises and during races, (4) its vendor, (5) the average running activity per week or per month, and (6) the number of running events in the last 12 months. The number of exercises and attended events was assumed as surrogate criterion to determine whether a participant was an amateur or (semi-)professional runner.

Q_2_ consisted of 5 items: (1) the tracked distance of (2) one or multiple devices, (3) sex, (4) course category, and (5) the starting block as given by the event organizer (see Multimedia Appendix 2 for questions and response options). Different starting blocks were used to determine whether a runner classified himself or herself as fast or slow.

Runners participated on a voluntary basis in the surveys. Neither personal data nor contact details were collected. Therefore, the resulting records were considered an anonymous dataset that did not conflict with the legislation of national or federal data privacy laws in Germany.

Runners could fill out the paper-based Q_1_ on their own. However, most of them preferred to be guided by our survey staff, which consisted of the authors and a group of 9 selected and well-briefed students. The interviewer staff checked whether potential survey candidates had already been asked to participate. Thus, the number of duplicate data entries could be kept very low. In case a participant actively declined an interview, no data at all were noted down.

For the post-race survey, randomly selected race finishers were interviewed. In order to prevent device misreadings caused by physical exhaustion, athletes were not allowed to fill out questionnaires on their own. Instead, their answers were put directly into the corresponding questionnaire by the survey staff.

### Recruitment

Only runners of more than the minimum participation age (>=16 years) were included in the Trollinger-Marathon cohort. Persons who took part in the marathon relay were excluded from the post-race survey, as no precise information about the relay course sections was made available by the organizers.

For the pre-race survey, the interviews were conducted on May 7 (11:30 AM till 6:30 PM) and May 8 (6:30 AM till 10:00 AM), 2016, while the runners picked up their number bibs, timing chips, and event information. The post-race survey was carried out on May 8 (11:45 AM till 2:15 PM), 2016, at the finish area located in the Heilbronn Frankenstadion.

### Data Exclusion

In case of inconclusive device or vendor information and illegible handwriting, questionnaires were strictly excluded, as well as questionnaires with missing information on tracked distances. Thus, for Q_1_ and Q_2_, the number of related dropouts were 2.7% (25/923) and 21.8% (73/335), respectively.

### Statistical Analysis

For further analyses, the remaining, valid questionnaires were transcribed into a relational database setup for this purpose; 1 person read the values as noted in Q_1_ and Q_2,_ whereas another person entered the data into a corresponding data entry mask. Next, the transcribed data were analyzed with the statistics software R version 3.1.2 (R Foundation for Statistical Computing, Vienna, Austria) [17].

Age and sex distributions of the study cohort were compared with the official event starter list—as provided by the organizer—to ensure a satisfying level of representativeness. Logistic regression analysis was applied to examine influencing factors such as sex, age, and exercise frequency on the prevalence of smart devices in the respective subcohorts.

### Analysis on Recorded Distance

In theory, the recorded distances should be comparable with each other, as both, the full and the half-marathon, were AIMS-certified for road races.

However, it is unlikely that the exact distance of 42.195 km and 21.0975 km is being recorded, as not every runner can follow the perfect racing line. Moreover, runners may change the road side, resulting in slightly longer distances. For this reason, it is not valid to compare the absolute deviations between the recorded distance and the official track distance (each in kilometers) as true value of the mean. Therefore, it is necessary to compare measured distances with each other via a 2-sided, 2-sample *t* test (significance level alpha=.05). The *t* test was applied to analyze differences among identified device categories, as presented in the following sections.

## Results

### Principal Findings

A total of 898 valid Q_1_ and 262 valid Q_2_ were collected and subsequently transcribed into the study database.

### Study Cohort

The cohort of the pre-race survey comprised 78.7% (133/169) male and 21.3% (36/169) female full marathon runners. For the half-marathon, 61.9% (396/635) males and 37.3% (239/635) females were recorded. According to the organizer’s starting list, 82.4% (593/720) of the marathon runners were males and 17.6% (127/720) female.

For the half-marathon course, a higher percentage of female runners (27.01%, 1492/5524) had themselves registered (male: 72.99%, 4032/5524). Table 1 shows the distribution of sex and age for the full and half-marathon.

For the walking or nordic walking course and the marathon relay event, 32 and 18 questionnaires were collected, respectively. For both subcohorts, no further breakdown for sex or age was conducted.

A total of 39 runners did not fill in the actual event type they took part in and were thus excluded from the cohort analysis.

**Table 1:** Distribution of sex and age groups among runners for the full and half-marathon (Q_1_).

Event	Age group (years)	Male	Pr_survey_ (%)	Pr_official_^a^(%)	Female	Pr_survey_ (%)	Pr_official_^a^(%)
Marathon							
	16-29	19	14.3	11	6	16.7	16
	39-39	23	17.3	21	10	27.8	27
	40-49	45	33.8	33	9	25.0	33
	50-59	36	27.1	28	11	30.6	23
	60-69	8	6.0	7	0	0.00	2
	70-79	2	1.5	1	0	0.00	0
	80+	0	0	0	0	0.00	0
	Unknown	0	0	0	0	0.00	0
	Total	133			36		
Half-marathon							
	16-29	94	23.7	22	66	27.6	30
	39-39	84	21.2	28	48	20.1	28
	40-49	93	23.5	25	68	28.5	23
	50-59	97	24.5	20	48	20.1	15
	60-69	24	6.1	5	9	3.8	3
	70-79	4	1.0	1	0	0	0
	80+	0	0	0	0	0	0
	Unknown	0	0	0.2	0	0	0.1
Total		396			239		

^a^Values in curved brackets (Pr_official_) denote the proportion as given in the official starter list for the respective subcohort.

During the post-race survey, questionnaires of 88% (38/43) male and 12% (5/43) female marathon runners and 82.5% (175/212) male and 17.5% (37/212) female half-marathon runners were collected; 2 runners did not state their sex. For the walking or nordic walking course, 5 questionnaires were collected.

### Device Category

According to the qualitative data on device names and respective vendor information collected via Q_1_ and Q_2_, the authors identified 6 major categories of devices: (D_1_) mobile phones with related app, (D_2_) GPS-enabled sport watches, (D_3_) heart rate monitors, (D_4_) smart watches, (D_5_) wristband activity trackers, and (D_6_) other devices.

However, technical differentiation among these categories is a difficult task. As of today some GPS-enabled sport watches can be paired with mobile phones and receive text messages or push notifications. In case the primary purpose of a device was the support of physical activities, it was classified into D_1_ rather than D_4._ For instance, the Apple Watch was classified in D_4_, as it was primarily a lifestyle device. A device was classified as wristband activity tracker if its general shape resembled a bracelet, for example, the Garmin vivofit or Polar Loop. Other Devices (D_6_) included simplistic GPS receivers, chest harnesses, GPS-enabled devices for golf court navigation, or even simple analog or digital watches. Device names and the number of occurrences are presented in Table 2. For reasons of clarity and comprehensibility, only devices that occurred 5 or more times in the dataset are listed (for a detailed table with all occurrences, see Multimedia Appendix 3).

**Table 2:** Device categories, vendors, models, and apps used by runners. Only vendors, devices, and apps with ≥5 occurrences collected with Q_1_ are listed. Values in curved brackets represent the number of mentions for the respective category, vendor, device, or app.

Category	Vendors	Devices
D_1_: Mobile phone and app (181)	Apple (80), Samsung (65), Sony (11)	iPhone 6 (22), iPhone 5s (19), iPhone 5 (12), iPhone (11), Galaxy S5 (11), Galaxy S4 (10), iPhone 6s (9), Galaxy S4 mini (8), Galaxy S3 (7), Samsung: other (7) Apps: Runtastic (126), Runkeeper (10), Nike+ Running (9), Endomondo (7), Sports Tracker (6), Strava (6)
D_2_: GPS sport watch (437 **)**	Garmin (193), Polar (165), TomTom (38), Suunto (18)	M400 (60), Garmin: other (41), V800 (31), Polar: other (31), Forerunner 305 (22), Forerunner 310XT (19), TomTom: other (16), Forerunner 920XT (13), Forerunner 610 (12), Runner Cardio (11), RS300X (10), Ambit 3 Peak (10), Fenix 3 (10), Forerunner 210 HR (9), RC3 (9), RS800CX (9), RCX5 (8), Garmin: other Forerunners (8), RCX3 (7), Forerunner 910XT HR (7), Forerunner 235 WHR (7), Forerunner 205 (6), Forerunner 220 (6), vivoactive (6), Forerunner 110 HR (5), other GPS-enabled sport watch (5)
D_3_: Heart rate monitor (37)	Polar (27)	Polar heart rate monitor: other (8), heart rate monitor: other (7), A300 (6)
D_4_: Smart watch (14)	Apple (12)	Apple Watch (12)
D_5_: Wristband activity tracker (27)	Garmin (11), Polar (8)	Loop (6), vivofit (5), vivosmart HR (5)
D_6_: Other devices (47)	No specific vendor (36)	Stopwatch (25), watch (6)

As given in Table 2, mobile phones sold by Apple and Samsung were predominant in the study cohort. The majority of the interviewed participants in the D_1_ category preferred Runtastic as an accompanying app (69.6%, 126/181), followed by other running apps such as Runkeeper or Nike+ Running. The GPS-enabled sport watch segment (D_2_) was also dominated by 2 vendors in particular: Garmin 44.2% (193/437) and Polar 37.6% (165/437). The most popular device was the Polar M400 13.7% (60/437). Devices in the category D_4_ (1.9%, 14/743) and D_5_ (3.6%, 27/743) seemed to be underrepresented among runners.

### Adoption of Wearable Technology

Results of the pre-race survey obtained by Q_1_ showed that 26.1% (234/898) of the runners did not use any device for their exercises or during a running event. In contrast, 8.8% (79/898) of the athletes stated that they used more than 1 device.

Given a total of 977 recorded devices 44.7% (437/977) represented GPS-enabled sport watches, and 18.5% (181/977) were mobile phones with a combined app to track the running performance. The proportion of heart rate monitors (3.8%, 37/977), smart watches (1.4%, 14/977), and wristband activity trackers (2.8%, 27/977) was quite low.

Regression analysis showed that the relation between females and higher age groups and no usage of additional devices for exercise was statistically significant (Table 3). The subcohort of runners with a higher exercise frequency seemed to be associated with the use of wearable devices for training optimization (odds ratio 2.627). However, this finding was not statistically significant.

**Table 3:** Features associated with wearable devices and training optimization or distance tracking (n=977).

Feature (n=977^a^)		Odds ratio	95% CI	*P* value
**Exercise**				
	Once a month (Ref^b^)	1.0		
	Once a week	0.629	0.028-7.040	.71
	Twice a week	1.590	0.072-17.274	.71
	Three times or more a week	2.627	0.119-28.466	.44
	No exercise	0.299	0.012-4.034	.38
**Sex**				
	Male (Ref^b^)	1.0		
	Female	0.673	0.486-0.933	.02
	Unknown	0.700	0.200-3.266	.61
**Age, years**				
	16-29 (Ref^b^)	1.0		
	30-39	1.127	0.693-1.838	.63
	40-49	1.009	0.641-1.584	.97
	50-59	0.607	0.385-0.949	.03
	60-69	0.312	0.159-0.617	<.001
	70-79	0.079	0.011-0.400	.004
**Event**				
	Half-marathon (Ref^b^)	1.0		
	Marathon	1.017	0.667-1.578	.94
	Marathon relay	1.891	0.596-8.430	.33
	Walking or nordic walking	0.781	0.372-1.690	.52
	Unknown	1.734	0.767-4.487	.22

^a^An extra of 79 data points is included due to multiple answers.

^b^Reference group in the regression model.

An analysis of device records for the full and half-marathon participants revealed that, in both groups, the majority of runners preferred GPS-enabled sport watches (full: 57.5%, 104/181; half: 42.6%, 297/698). Interestingly, the usage of mobile phones in combination with running apps was more prevalent for half-marathon participants (full: 12.2%, 22/181; half: 19.5%, 136/698).

### Accuracy of Tracking Devices

In total, 270 track distances were collected in the post-race survey. Some devices recorded both the number of tracked kilometers and the number of footsteps. The majority of measurements was given in kilometers (97.0%, 262/270). The average number of kilometers for the full marathon and half-marathon courses was 42.385 and 21.154 km, respectively. Table 4 shows the mean recorded distances for each device category, in case the devices were equipped with sensors to track distances.

**Table 4:** Mean, median, and I and II quartiles of the recorded distances for the full and half-marathon. Median and quartiles are not reported for categories that had <10 data points.

Marathon^a^	n	Mean (km)	Median (km)	Quartile I (km)	Quartile II (km)
D_1_: Mobile phone and app	4	42.88	–	–	–
D_2_: GPS sport watch	39	42.33	42.29	42.20	42.38
Half-marathon^a^					
D_1_: Mobile phone and app	30	21.40	21.41	21.13	21.55
D_2_: GPS sport watch	179	21.18	21.17	21.09	21.23
D_4_: Smart watch	2	21.2	–	–	–
D_5_: Wristband activity tracker	3	20.38	–	–	–

^a^D_3_ and D_6_ devices were technically not equipped with tracking sensor technology.

The longest recorded distances were 43.7 km (full) and 22.55 km (half) and the shortest 41.48 km (full) and 20.00 km (half), respectively; that is, the maximal deviations were 1.5 km for the full marathon and 1.45 km for the half-marathon course. The minimal deviations for both courses were found for the GPS-enabled sport watches. With a mean absolute error (MAE) of 0.35 km (1.7%), mobile phones (D_1_) slightly overestimated the half-marathon course. In contrast, measurements obtained by GPS-enabled sport watches (D_2_) showed a smaller MAE of 0.12 km (0.6%).

As outlined in Table 4, the number of collected samples for D_4_ and D_5_ as well as the number of full marathon samples (n=43) was too small. For this reason, only the remaining 2 groups (D_1_ and D_2_) could be tested in the half-marathon group. In terms of difference in mean, half-marathon measurements collected for mobile phones (D_1_) and sport watches (D_2_) were not equal to each other (*P*=.002).

**Figure 1:**
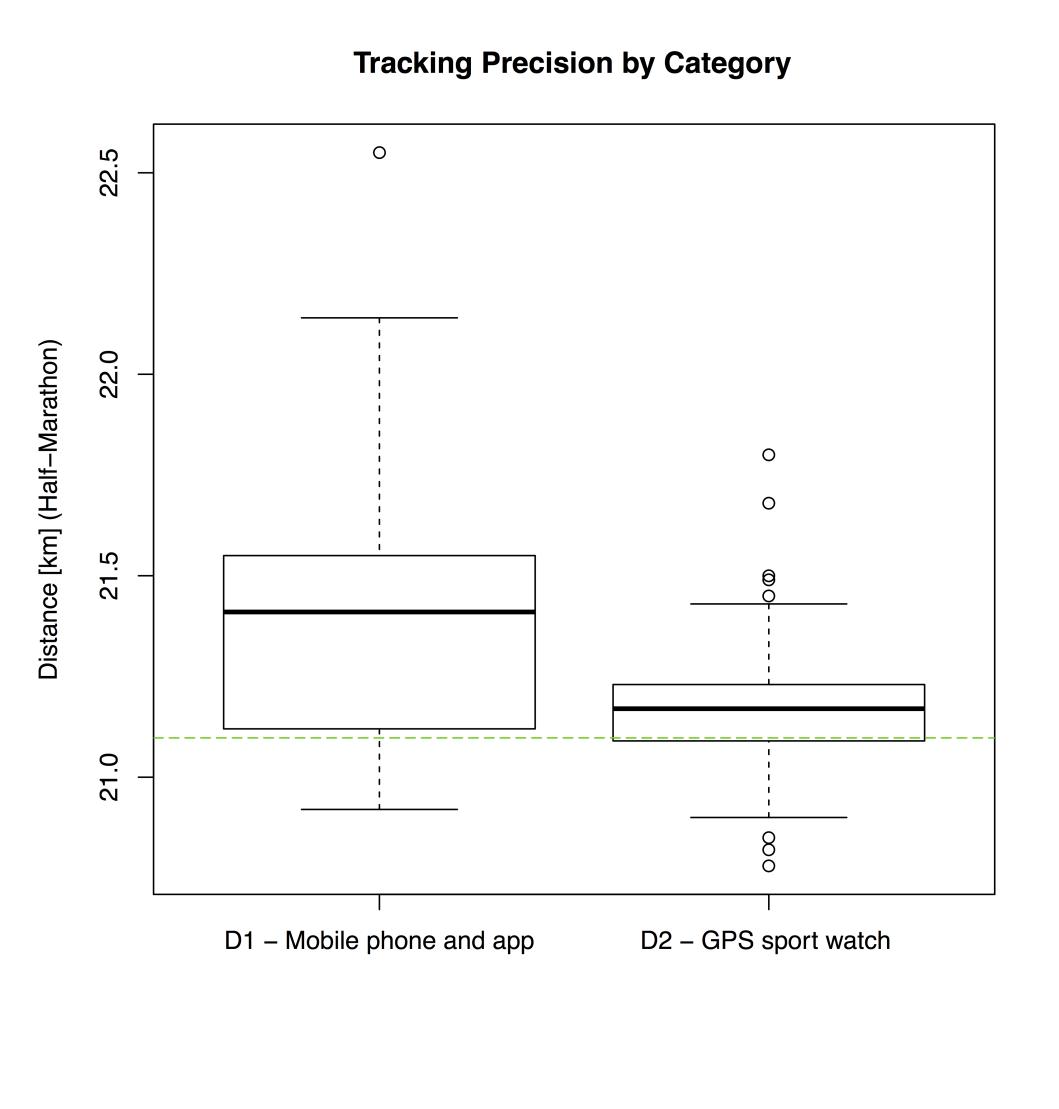
Box-and-whisker plot of recorded distances (half-marathon) by device category D1 (n=30) and D2 (n=179). The dashed line indicates the reference distance of 21.0975 km.

**Figure 2:**
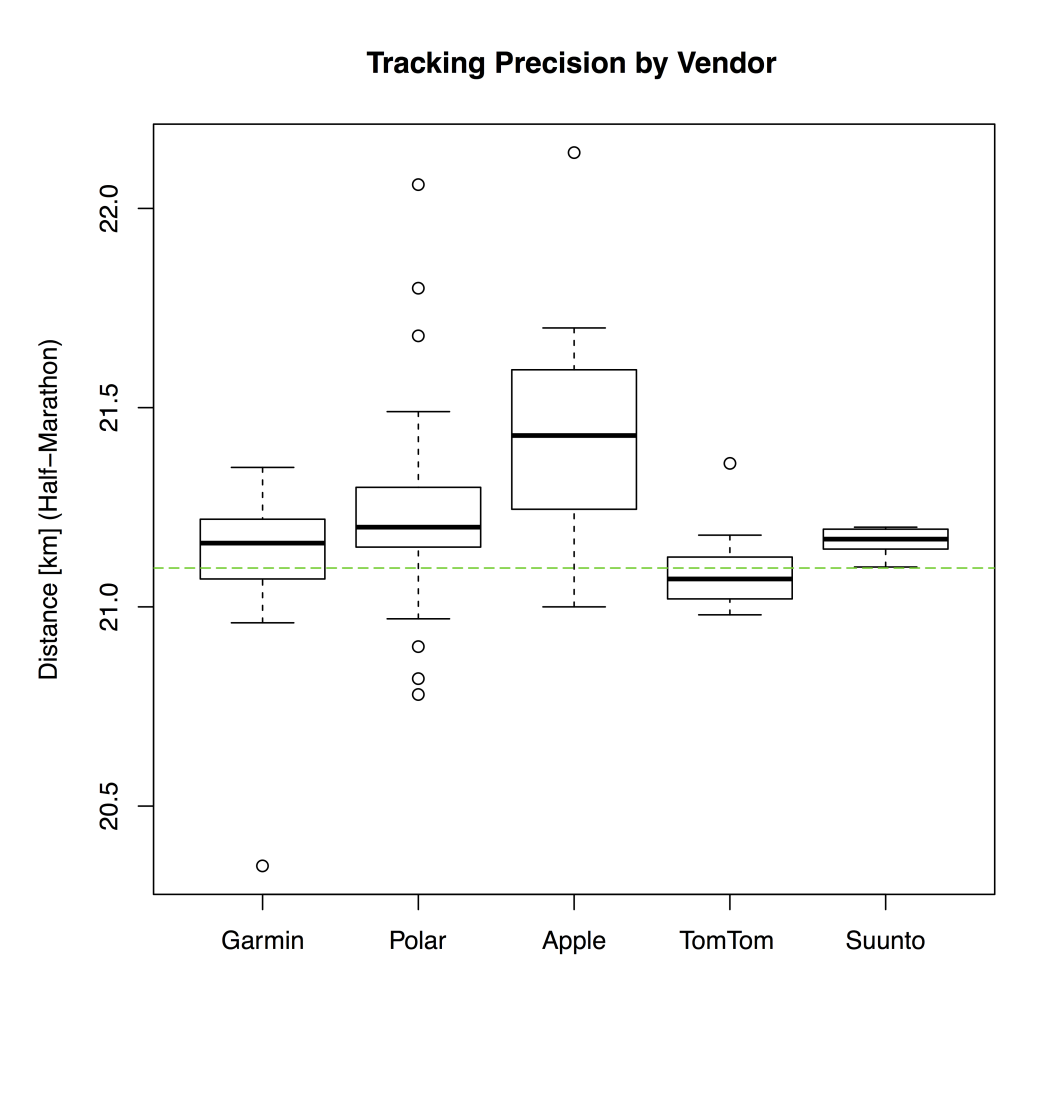
Box-and-whisker plot of recorded distances (half-marathon) by vendor: Garmin (n=77), Polar (n=72), Apple (n=20), TomTom (n=16), and Suunto (n=7). Vendors with less than 7 measurements were omitted. The dashed line indicates the reference distance of 21.0975 km.

**Figure 3:**
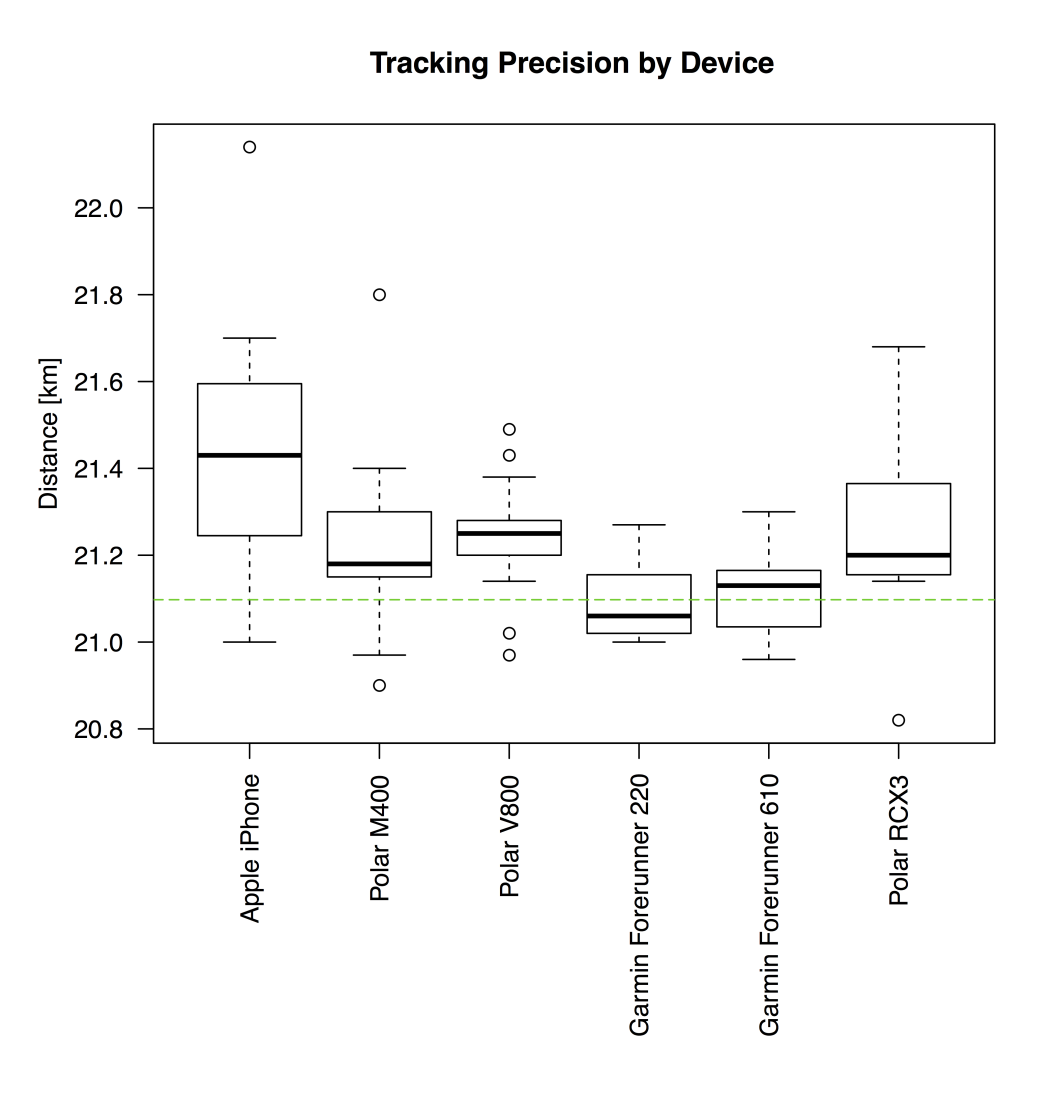
Box-and-whisker plot of recorded distances (half-marathon) by device: Apple iPhone (n=20), Polar M400 (n=36), Polar V800 (n=21), Garmin Forerunner 220 (n=7), Garmin Forerunner 610 (n=7), and Polar RCX3 (n=7). Devices with less than 7 measurements were omitted. The dashed line indicates the reference distance of 21.0975 km.

For further analysis of half-marathon data, the aforementioned categories, vendors, and devices were compared against each other, visualized via 3 box-and-whisker plots, as depicted in Figures 1-3.

Measurements for devices in D_1_ showed a higher variance as devices in D_2_, which corresponded to the result of the *t* test and findings in Table 4.

Figures 2 and 3 give a more detailed breakdown for different vendors and frequently used devices at the Trollinger-Marathon. The interquartile ranges (IQRs) by Garmin and Polar devices are comparable. However, data generated by Polar devices show a higher number of statistical outliers. The IQR of TomTom and Suunto devices was found to be the lowest, yet it must be noted that only 16 and 7 data points were available. As depicted in Figure 3, the Garmin devices seem to be the most accurate against the reference distance of the half-marathon course. In contrast, measurements of mobile phones (here: Apple iPhone) show the highest IQR and noticeably deviate from the reference distance (indicated by a dashed line).

## Discussion

### Principal Findings

There is evidence that “smart” devices such as smart watches and activity trackers are not as prevalent in the runners’ community as one might assume according to recent trend surveys regarding wearable usage [1,2].

Our results indicated that conventional GPS-enabled sport watches were predominant for a diverse population of active runners of different fitness levels.

A corresponding logistic regression analysis suggested that supportive technology was not associated with female persons and persons of higher age groups (60+ years). These findings corresponded with studies on mobile phone ownership, indicating that persons of younger age groups (18-49 years) are more likely to own a mobile phone [18].

The recorded data of GPS-enabled sport watches (D_2_) showed the highest accuracy with an average of 42.33 km (full marathon) and 21.18 km (half-marathon). The data captured with mobile phones in combination with an app (D_1_) were also quite accurate (average of 42.88 km and 21.40 km). All other relevant device categories D_4_ and D_5_, that is, smart watches and wristband activity trackers, were not tested due to a limited sample size.

Overall, the IQR was smaller for GPS-enabled sport watches (D_2_) than for mobile phones with combined app (D_1_). Measurements of mobile phones showed the highest IQR and noticeably deviated from the reference track distance.

The collected pre-race questionnaires for the full and half-marathon events were a representative sample for the persons that registered for the Trollinger-Marathon Event 2016. The distribution of age groups and sex in the sample was very similar to the proportions reported in the official starter lists.

### Limitations

This study suffered from several limitations. As the Trollinger-Marathon 2016 was a regional road race event, only runners from southern Germany were represented in the data of the two survey parts. Yet, no studies exist that show a regional difference in terms of technology affinity in Germany. Therefore, the authors are confident that the results of the survey could be applied to other German regions or road race events as well. However, the results of the Trollinger-Marathon study should be reproduced in other regions and countries to confirm the results. Moreover, as external parameters such as temperature and relative humidity were influencing factors to runners [19,20] and potentially their motivation to participate, it could not be ruled out that the cohort population might be different in another environmental setting, for example, during another season or climate zone.

Furthermore, no explicit checks for duplicate data acquisition were conducted by the interviewer team during the survey. This originated from the fact that most of the event participants were only available for less than a minute when fetching their number bibs and event information. Additionally, due to data privacy aspects, no names or contact information was written down. Thus, a check for duplicates was not possible for obvious reasons. The authors were confident that only a very low number of duplicate data entries occurred.

Participants quickly left the finish area after the event, resulting in a narrow time frame for the interviewers. Therefore, the study suffered from a comparatively small sample size for the post-race questionnaires. Moreover, a higher amount of runners declined to take part in the survey, as most of them were exhausted. As a consequence, the accuracy analysis could not be conducted for the categories D_4_ and D_5_ due to a small sample size for these particular devices. This experience indicates that the amount of time spent for interviews during a running event should be kept as minimal as possible. However, this restricts the possibilities for qualitative approaches.

In the pre-race phase only, 32 questionnaires for the (nordic) walking event could be collected. A reason for this low response rate was that a major fraction (according to the starter list: 56.5%, 345/611) of the registered walkers or nordic walkers were employees of the main sponsors of the event and the handout of number bibs and event information was conducted at a different on-site location for these participants, which was not accessible for the interview staff.

### Comparison With Prior Work

Several studies on the validity and accuracy of consumer-level devices, wearables, or mobile phones, especially pedometers or accelerometer-based technology, exist [10,20-25]. These studies are mostly laboratory based and do not collect data from participants of a running event. Instead, study subjects are equipped with (several) tracking devices, strictly following a study protocol for different types of exercises, for example, treadmill exercises.

In 2014, a meta-review by Bort-Roig et al analyzed whether mobile phone technology was suited for physical activity monitoring. The authors found only a “few studies” that reported on the validity of mobile phone–based assessment. However, “those that did report on measurement properties found average-to-excellent levels of accuracy for different behaviors” [26].

A study on mobile phone pedometers by Leong et al investigated on the reliability of free pedometer Android-based apps (Runtastic, Pacer Works, and Tayutau). They tested whether pedometer-apps were as accurate as a reference pedometer in a free-living environment for 7 days. The authors concluded that “none of the pedometer apps counted steps accurately compared to the reference pedometer,” (p.6) [25].

The studies by Tucker et al [27] and Hendelman et al [20] focused on the validity of step counts and the evaluation of estimated energy expenditure. In contrast, the evaluation of energy consumption was not part of the Trollinger-Marathon study.

In the late 1990s, Schutz et al [28] assessed GPS-based distance recording and found “the GPS technique (...) very promising.” Later research by Maddison et al [29], Cummins et al [30], and Larsson [31] confirmed these findings. For sport-specific field testing, the differential global positioning system (dGPS) was found to have an “acceptable precision” [32]. This was confirmed with the tracking data of GPS-enabled devices observed in the Trollinger-Marathon cohort. All aforementioned studies recruited only around 10-44 participants in their respective study cohort, whereas this study relied on 262 distance data points. In addition, our work referred to a long, precisely measured running course and might therefore be considered a real-world wearable technology evaluation. The general user acceptance and related use pattern was investigated by Shih et al, yet ”research focuses mostly on the technical- or device-related challenges” and “less research has focused on individual-related use and adoption challenges” (p.4) [32].

Work by Mauriello et al [33] evaluated a wearable e-textile display with various runners (n=52). The authors reported that their cohort also favored wearable devices by Garmin. Moreover, they found a similar proportion of runners who used no supportive “smart” technology during training sessions: “11 participants (21%) reported using pen and paper” compared with 26.1% in our cohort.

To the best knowledge of the authors, no work on the adoption of wearable technology for long-distance running activities exists in the literature. This study adds first answers to the question which devices are being used by healthy and active citizens of different sex, age, and fitness level participating in half-marathon and marathon events (including nordic walking and walking).

### Conclusions

Most of the runners (approximately 75%) who attended an official road running event in southern Germany used wearable technology for training optimization and distance recording. However, the findings of the study indicate that female runners and runners of higher age groups (60+ years) are less likely to use tracking devices for personal running activities.

With 156 identified distinct devices, 25 running apps, and 36 different vendors, the survey revealed that a great variety of wearable or smart technology was actively used by the cohort. Sport watches represented more than 65.4% of all devices of the study. GPS-enabled devices (sport watches and mobile phones) were found to be accurate in terms of recorded course distances. Yet, the mean of recorded distances between sport watches and mobile phones in combination with apps was significantly different for the half-marathon course (*P*=.002). However, given a long-distance running event, an MAE of 0.12 km (sport watch) versus 0.35 km (mobile phone and app) seems negligible, as this corresponds to approximately 0.6%-1.7% of the total course distance.

To validate our findings, we intend to repeat the study at the next edition of the Trollinger-Marathon (in 2017). Such a follow-up study might confirm adoption rates in 2016 or discover a shift of wearable technology use by runners.

## Appendix

Multimedia Appendix 1 Questions and response options of the pre-race questionnaire. This is a translation of the original questionnaire in German language.

Multimedia Appendix 2 Questions and response options of the post-race questionnaire. This is a translation of the original questionnaire in German language.

Multimedia Appendix 3 Device categories, vendors, models and apps used by runners as found in the pre-race survey. Values in curved brackets represent the number of occurrences for the respective category, vendor, device or app.

## Abbreviations

AIMS: Association of International Marathons and Road Races
dGPS: differential global positioning system
GPS: global positioning system
IAAF: International Association of Athletics Federation
IQR: interquartile range
MAE: mean absolute error
OR: odds ratio
Q1: pre-race questionnaire
Q2: post-race questionnaire
