# Supplementary material for: Accuracy and Adoption of Wearable Technology Used by Active Citizens: A Marathon Event Field Study
Source: JMIR Mhealth Uhealth. 2017 Feb 28;5(2):e24. doi: 10.2196/mhealth.6395 (PMC5350460; doi:10.2196/mhealth.6395)
Supplement: Multimedia Appendix 1 [file mhealth_v5i2e24_app1.pdf]

Multimedia Appendix 1: Pre-race questionnaire Q_1

Questions and response options of the pre-race questionnaire. This is a translation of the original questionnaire in German language.

Question	***Response options***
Which kind of device do you use for exercising or during running events?	Mobile phone and app<br>Sport watch<br>Smart watch<br>Wristband activity tracker<br>Other<br>None<br><br>+ open text for vendor and device/app name
What is your sex?	Male<br>Female
What is your age?	16-29<br>30-39<br>40-49<br>50-59<br>60-69<br>70-79<br>80+
How often do you exercise?	Once a week<br>Twice a week<br>Three times or more times a week<br>Once a month<br>Twice a month<br>No frequent exercises at all
In which event do you participate in?	Half-Marathon<br>Marathon<br>Walking
In the past twelve months, how many official running events did you participate in?<br>(Trollinger-Marathon 2016 excluded)	Yes<br>+ number of events<br><br>None
