# Supplementary material for: Accuracy and Adoption of Wearable Technology Used by Active Citizens: A Marathon Event Field Study
Source: JMIR Mhealth Uhealth. 2017 Feb 28;5(2):e24. doi: 10.2196/mhealth.6395 (PMC5350460; doi:10.2196/mhealth.6395)
Supplement: Multimedia Appendix 2 [file mhealth_v5i2e24_app2.pdf]

Multimedia Appendix 2: Post-race questionnaire Q_2

Questions and response options of the post-race questionnaire. This is a translation of the original questionnaire in German language.

Question	***Response options***
Which kind of device did you use?	Mobile phone and app<br>Sport watch<br>Smart watch<br>Wristband activity tracker<br>Other<br>None<br><br>+ open text for vendor and device/app name
How did the device record the distance?	Kilometers<br>Steps
What distance was tracked by your device?	Kilometers / number of steps
What is your sex?	Male<br>Female
In which starting block did you start?	Red<br>Blue<br>Yellow<br>Green<br>Walking
In which event did you participate in?	Half-Marathon<br>Marathon<br>Walking
