# Supplementary material for: Accuracy and Adoption of Wearable Technology Used by Active Citizens: A Marathon Event Field Study
Source: JMIR Mhealth Uhealth. 2017 Feb 28;5(2):e24. doi: 10.2196/mhealth.6395 (PMC5350460; doi:10.2196/mhealth.6395)
Supplement: Multimedia Appendix 3 [file mhealth_v5i2e24_app3.pdf]

## Multimedia Appendix 3: Device categories, vendors, models and apps

Device categories, vendors, models and apps used by runners as found in the pre-race survey. Values in curved brackets represent the number of occurrences for the respective category, vendor, device or app.

| *Category* | *Vendors* | *Devices* |
|---|---|---|
| | | |
| **$D_1$ –** Mobile phone and app (181) | Apple (80), Samsung (65), Sony (11), HTC (4), Google (4), Nokia (4), LG (4), Huawei (4), Motorola (1), Mobistel (1), BQ (1), Blackberry (1), alcatel (1) | iPhone 6 (22), iPhone 5s (19), iPhone 5 (12), iPhone (11), Galaxy S5 (11), Galaxy S4 (10), iPhone 6s (9), Galaxy S4 mini (8), Galaxy S3 (7), Samsung - other (7), Galaxy S5 mini (4), Galaxy S6 (4), Galaxy S2 (3), iPhone 4s (3), Xperia (3), Galaxy S3 mini (3), Nexus (3), iPhone 4 (3), Galaxy S7 (2), Xperia Z5 (2), LG - other (2), Huawei - other (2), Xperia Z3 (2), Lumia (2), Galaxy S6 Edge (2), Xperia Z5 Compact (2), One (2), Y530 (1), Sony - other (1), iPhone 6s Plus (1), Lumia 950XL (1), Galaxy Ace 2 (1), Blackberry (1), Nokia - other (1), Onetouch (1), LG L5 (1), Nexus 5 (1), Xperia Z1 (1), Galaxy Note 4 (1), Razr (1), LG G4 (1), Desire (1), One X+ (1), P7 (1), Galaxy Express (1), aquaris M5 (1), A3 (1), Mobistel E (1)<br><br>Apps:<br>Runtastic (126), Runkeeper (10), Nike+ Running (9), Endomondo (7), Sports Tracker (6), Strava (6), Runtastic Pro (4), Polar Flow (3), Noom Coach (2), adidas miCoach (1), Apple Health (1), Garmin Connect (1), Jabra Sport Life (1), KeepRunning (1), komoot (1), MapMyRun (1), My Asics (1), My Fitness App (1), MyTracks (1), Polar beat (1), Runmeter (1), S Health (1), Suunto Movescount (1) |
| **$D_2$ –** GPS sport watch (437**)** | Garmin (193), Polar (165), TomTom (38), Suunto (18), Other vendor (5), Fitbit (4), Sigma (3), Crane (2), Decathlon (2), Epson (2), Runtastic (2), A-Rival (1), Timex (1), adidas (1) | M400 (60), Garmin - other (41), V800 (31), Polar - other (31), Forerunner 305 (22), Forerunner 310XT (19), TomTom – other (16), Forerunner 920XT (13), Forerunner 610 (12), Runner Cardio (11), RS300X (10), Ambit 3 Peak (10), Fenix 3 (10), Forerunner 210 HR (9), RC3 (9), RS800CX (9), RCX5 (8), Garmin – other Forerunners (8), RCX3 (7), Forerunner 910XT HR (7), Forerunner 235 WHR (7), Forerunner 205 (6), Forerunner 220 (6), vivoactive (6), Forerunner 110 HR (5), other GPS-enabled sport watch (5), Forerunner 225 (4), Ambit 2 S HR (4), Suunto - other (4), Surge (4), Nike+ SportWatch (4), Sigma - other (3), |

| | | |
|---|---|---|
| | | Multisport (3), Forerunner 230 (3), Forerunner 410 HR (3), Forerunner 620 (3), Cane GPS-watch 2013 (2), Kalenji GPS-Sport watch (2), Runsense SF-810 (2), Forerunner 405 (2), Runtastic Heart Rate GPS Watch (2), Runner 2 Cardio (2), Fenix 2 (2), Runner (2), miCoach Smart Run (1), Forerunner 201 (1), SQ100 (1), Forerunner 10 (1), Forerunner 25 (1), Ironman Lauftrainer 2.0 (1), Fenix (1), Forerunner 630 HR (1) |
| **$D_3$ –** Heart rate monitor (37) | Polar (27), Other (7), Timex (1), Crivit (1), Sigma (1) | Polar heart rate monitor - other (8), heart rate monitor - other (7), A300 (6), RS100 (4), RS200sd (3), S720i (1), Ironman Race Trainer Pro (1), Crivit Heart rate Monitor (1), FT4 (1), RS400 (1), M91 ti (1), FT7 (1), Heart rate monitor RC14.11 (1), FT40 (1) |
| **$D_4$ –** Smart watch (14) | Apple (12), Samsung (1), Sony (1) | Apple Watch (12), SmartWatch 3 (1), Gear S2 (1) |
| **$D_5$ –** Wristband activity tracker (27) | Garmin (11), Polar (8), Fitbit (4), Epson (1), Other (1), Jawbone (1), Mio (1) | Loop (6), vivofit (5), vivosmart HR (5), Charge HR (3), A360 (2), Flex (1), UP2 (1), PS-500 (1), FUSE (1), Garmin Wristband Activity Tracker - other (1), Wristband Activity Tracker - other (1) |
| **$D_6$ –** Other devices (47) | No specific vendor (36), Garmin (4), Polar (4), Casio (1), Jabra (1), Fitbit (1) | Stopwatch (25), watch (6), mp3-Player (4), M450 (2), Chest Strap (2), Edge 510 (1), Pedometer (1), Approach G10 (1), Foretrex 401 (1), Casio Watch (1), One Activity- and Sleep-Tracker (1), Oregon 300 (1), Sport Pulse Earbuds (1) |
